# Potential Influential Factors of In-Hospital Myocardial Reinfarction in ST-Segment Elevation Myocardial Infarction (STEMI) Patients: Finding from the Improving Care for Cardiovascular Disease in China- (CCC-) Acute Coronary Syndrome (ACS) Project

**DOI:** 10.1155/2021/9977312

**Published:** 2021-10-06

**Authors:** Xiaojie Cai, Juan Zhou, Wenyuan Li, Lele Cheng, Zuyi Yuan, Yihui Xiao

**Affiliations:** Department of Cardiovascular Medicine, First Affiliated Hospital of Xi'an Jiaotong University, Xi'an, China

## Abstract

In this study, 39915 inpatients with a discharge diagnosis of STEMI from the CCC-ACS project phase I and II were included. The prevalence of the medical history, clinical complications on admission and treatment during hospitalization in the STEMI inpatients with and without in-hospital reinfarction was presented. The factors that were differentially distributed and of critical clinical significance (e.g., age, sex, heart rate, smoking, MI history, HF history, COPD history, stroke, hypertension, diabetes, PCI treatment, administration of DAPT, and statins) were entered into standard Cox regression model and competing risk model for potential influential factors of in-hospital reinfarction. Patients with a higher heart rate (OR 1.018; 95% CI 1.003 to 1.033) were more susceptible to in-hospital reinfarction. Myocardial infarction history (OR 2.840; 95% CI 1.160 to 6.955) was a risk factor of in-hospital reinfarction independent of hypertension, diabetes, and dyslipidaemia.

## 1. Introduction

Cardiovascular disease (CVD) remains a public health concern, causing death in 40% of the Chinese population [1]. Acute coronary syndrome (ACS), especially ST-segment elevation myocardial infarction (STEMI), is the most devastating clinical manifestation of CVD [2–5]. The prognosis of STEMI varies among individuals [6–9]. Reinfarction, major adverse cardiovascular event (MACE), sharply increases mortality among STEMI patients [10, 11]. In previous studies, Steen et al. tried to identify the risk factors of myocardial reinfarction after anaesthesia and surgery by analysing 587 patients during 1974 and 1975 at their institution who had a myocardial infarction history and suggested that postoperative intensive care unit admission did not significantly affect the reinfarction rate, nor did diabetes, angina, patient age, sex, or site of the previous myocardial infarction [12]. A randomized controlled trial with a factorial design was performed to examine the effects of dietary intervention in the secondary prevention of myocardial infarction (MI) and stated that the 2-year incidence of reinfarction plus death from ischaemic heart disease was not significantly affected by any of the dietary regimens [13]. Recently, some studies have defined reinfarction as a main outcome measure and tried to explore the relation between treatment and reinfarction [14–16]. However, few studies have focused on this high-risk population and performed a systematic evaluation.

People in China started paying attention to medical care for CVD in the 1970s [17]. Over the past decades, clinicians and epidemiologists have been trying to improve and standardize care for cardiovascular disease [18, 19]. In 2014, to estimate the prevalence and improve care for cardiovascular disease in China, the American Heart Association (AHA) and Chinese Society of Cardiology (CSC) launched a nationwide registry and quality improvement study in China—Care for Cardiovascular Disease in China (CCC) project. A total of 150 tertiary hospitals from 30 provinces were included in this project, which consists of the acute coronary syndrome (CCC-ACS) project and atrial fibrillation (CCC-AF) project. From November 2014 to June 2017, 63641 ACS inpatients were registered in the CCC-ACS project. Details of the design and methodology of the CCC project have been published previously [20].

**Figure 1:**
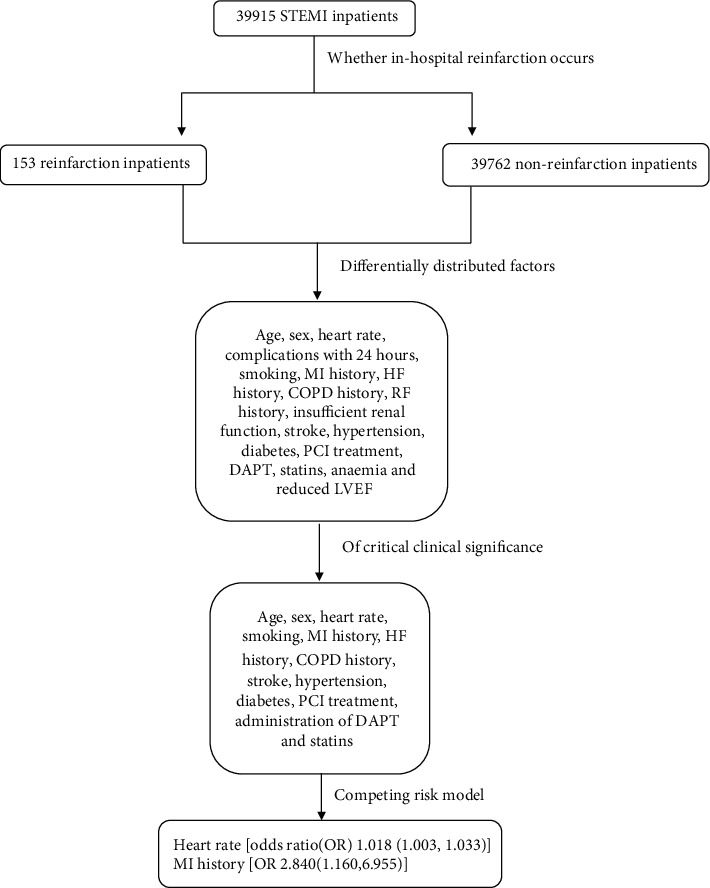
Workflow.

Data from 39915 STEMI patients participating in the CCC-ACS project were used in this study. We first provide the prevalence of medical history and clinical complications on admission and treatment during hospitalization of the STEMI inpatients with and without in-hospital reinfarction. The factors that were differentially distributed and of critical clinical significance were entered into standard Cox regression and competing risk models to identify potential influential factors of in-hospital reinfarction (Figure 1: workflow). The study benefited from the large population size and appreciable geographical distribution within China. We think that the data we showed in this study comprehensively fill a gap in the knowledge about the in-hospital reinfarction population.

## 2. Study Design and Methods

Institutional review board approval was granted for the aggregate data set for research and quality improvement by the Ethics Committee of Beijing An Zhen Hospital, Capital Medical University. Participating sites were granted a waiver of patient consent under the common rule. Thirty-nine sites received institutional review board approval from their own ethics committees, with the other 111 sites accepting central ethics approval. The study is registered at http://www.clinicaltrials.gov/ (NCT02306616).

### 2.1. Study Population

The CCC-ACS project, whose aim is to improve and evaluate the quality of care for ACS inpatients, is a nationwide quality improvement registry programme launched in 2014. Hospitals were recruited to represent the diversity of ACS care in tertiary hospitals in different geographic-economic regions in China. In each geographical region of the seven regions in China: Northern, Northeast, Eastern, Central, Southern, Southwest, and Eastern China, provinces are grouped into quartiles according to gross domestic product per capita, namely, low, medium-low, medium-high, and high levels. In each geographic-economic region, 10% of the tertiary hospitals were recruited for our study, with 150 hospitals selected in phases I and II. The rationale and design of the study have been published previously. Briefly, from the 150 recruited tertiary hospitals in phases I and II, the first 20 to 30 inpatients at each hospital were consecutively recruited for the CCC-ACS project. In this study, we included inpatients with a discharge diagnosis of STEMI. STEMI was defined strictly in compliance with the guidelines issued by the CSC for diagnosis and management [21–23]. The diagnostic criteria for STEMI were based on chest pain or discomfort, electrocardiogram (ECG), and measurements of myocardial injury biomarkers. From November 2014 to June 2017, 39915 STEMI inpatients were registered in the CCC-ACS project phases I and II.

### 2.2. Data Collection

In this study, a standard web-based data collection platform (Oracle Clinical Remote Data Capture, Oracle) was applied for trained data abstractors at the participating hospitals to report the required data from original medical records. Each month, eligible patients were consecutively reported to the CCC-ACS database before the middle of the following month. The data have been made available for onsite audits by third parties for quality control. According to the quality audit reports, the data in this study were properly reported with an accuracy rate greater than 95%. Data elements collected in this study included patients' demographics, medical history, symptoms on arrival, in-hospital treatments and procedures, discharge medications, and secondary prevention strategies.

### 2.3. Definition of STEMI

STEMI is defined as persistent chest discomfort or other symptoms suggestive of ischaemia and ST-segment elevation in at least two continuous leads [4, 24].

### 2.4. Definition of In-Hospital Reinfarction

We defined in-hospital myocardial infarction as a second acute myocardial infarction during hospitalization in STEMI inpatients, including reinfarction and recurrent infarction. Time to event was defined as the time difference between (1) the date of reinfarction and the date of admission for reinfarction patients, (2) the date of death and the date of admission for patients who died during this hospitalization, and (3) the date of hospital discharge and the date of admission for those who had neither of them [25].

### 2.5. Study Variables

Admission complications were defined as the symptoms complicating this attack within 24 hours, including cardiac shock (CS), heart failure (HF), and cardiac arrest (CA). Current smoking was defined as smoking in the last year. Diabetes was defined as a medical history of diabetes or use of glucose-lowering therapy before hospitalization or having a fasting blood glucose level ≥ 7.0 mmol/L (126 mg/dL) or glycated haemoglobin A1c (HbA1c) concentration ≥ 6.5%. Hypertension was defined as having a history of hypertension, use of antihypertensive therapy, or systolic blood pressure (SBP) ≥ 140 mmHg or diastolic blood pressure (DBP) ≥ 90 mmHg at admission. Dyslipidaemia was defined as a history of dyslipidaemia or serum low-density lipoprotein cholesterol (LDL‐C) ≥ 1.8 mmol/L (70 mg/dL) or serum high-density lipoprotein cholesterol (HDL‐C) < 1.0 mmol/L (40 mg/dL) or serum triglyceride (TG) ≥ 2.3 mmol/L (200 mg/dL). Insufficient renal function was defined as an estimated glomerular filtration rate < 60 mL · min^−1^ · 1.73 m^−2^. Anaemia was defined as having haemoglobin < 120 g/L for males and <110 g/L for females. Reduced left ventricular ejection fraction (LVEF) was defined as having an LVEF < 50%. A history of coronary heart disease (CHD) was defined as a history of MI or percutaneous coronary intervention (PCI) or coronary artery bypass grafting (CABG) before the current hospitalization. The transfer status indicated whether the patient was transferred from another hospital. Cerebrovascular disease, heart failure (HF), peripheral artery disease (PAD), atrial fibrillation (AF), and renal failure (RF) were defined according to the notes on original medical records. Acute management included fibrinolysis, PCI, and medicine given within 24 hours on admission via oral/intravenous administration, including dual antiplatelet therapy (DAPT), anticoagulants, statins, *β*-blockers, and angiotensin-converting enzyme inhibitors (ACEIs)/angiotensin-receptor blockers (ARBs) [26].

### 2.6. Statistical Analysis

Continuous variables with a normal distribution were shown as the mean (standard deviation (SD)), and differences between groups were compared using *t*-tests; continuous variables with a skewed distribution were shown as the median (interquartile range (IQR)) and were compared using the Mann–Whitney *U* test; categorical variables were presented as the percentage (number) and were compared using the chi-square test. Baseline characteristics were described and included vital signs, symptoms at arrival, medical history, and treatment during hospitalization, i.e., age (continuous), sex (male/female), systolic blood pressure (SBP; continuous), diastolic blood pressure (DBP; continuous), heart rate (HR; continuous), cardiac complications on admission (yes/no), Killip class on admission (class I/II–III/IV), current smoking (yes/no), history of MI (yes/no), HF (yes/no), chronic obstructive pulmonary disease (COPD; yes/no), cerebrovascular disease (stroke; yes/no), peripheral artery disease (PAD; yes/no), renal failure (RF; yes/no), administration of DAPT (yes/no), anticoagulant (yes/no), statins (yes/no), *β*-blockers (yes/no), ACEIs/ARBs (yes/no) during hospitalization, PCI treatment (yes/no), and whether patients were transferred from another hospital before the current hospitalization (yes/no).

We used a standard Cox survival regression and a competing risk regression that considered in-hospital death as a competing risk event to address potential risk factors (e.g., age, sex, heart rate, smoking, MI history, HF history, COPD history, stroke, hypertension, diabetes, PCI treatment, administration of DAPT, and statins) of in-hospital reinfarction (the framework can be seen in Figure 1).

Statistical analyses were performed using SPSS 22.0 (IBM, USA) and Stata 14.2 (Stata, College Station, TX, USA). Two-tailed *p* values of less than 0.05 were considered indicative of statistical significance.

## 3. Results

### 3.1. Patient Characteristics

Among the 39915 STEMI inpatients registered in the CCC-ACS project, 153 experienced in-hospital myocardial reinfarction. The medical characteristics on admission are presented in Table 1. In-hospital reinfarction increases the mortality rate from 2.2% to 30.7%. The reinfarction population had an average age of 67.60 ± 11.1 years old, and 34% were women. In the STEMI patients who did not experience reinfarction in the hospital, the average age was 61.63 ± 12.6 years old, and the proportion of women was 21.7%. A higher heart rate was observed in reinfarction patients (82.94 ± 17.9 vs. 77.96 ± 16.5 bpm, *p* < 0.001). The prevalence of severe clinical complications, including HF (18.0% vs. 8.9%, *p* < 0.001), CS (11.8% vs. 4.1%, *p* < 0.001), and CA (7.9% vs. 2.6%, *p* < 0.001), was higher in the reinfarction patients. With regard to cardiac function on admission, 60% of the patients with reinfarction were in Killip class I, 28.0% were in II-III, and 12% were in IV; among patients without in-hospital reinfarction, 70.6% were in I, 24.8% were in II-III, and 4.6% were in IV.

In terms of medical history, reinfarction patients had a higher prevalence of MI history (20.3% vs. 5.2%, *p* < 0.001), HF history (5.9% vs. 0.9%, *p* = 0.001), COPD history (4.6% vs. 1.3%, *p* = 0.001), RF history (3.9% vs. 1.1%, *p* = 0.001), stroke (14.4% vs. 8.6%, *p* = 0.012), hypertension (75.2% vs. 64.1%, *p* = 0.002), diabetes (56.4% vs. 47.0%, *p* = 0.028), anaemia (32.6% vs. 19.8%, *p* < 0.001), and reduced LVEF (50.9% vs. 31.2%, *p* < 0.001). Moreover, the proportions of CHD family history (1.3% vs. 2.5, *p* = 0.481), atrial fibrillation history (2.0% vs. 1.5%, *p* = 0.856), valvular disease history (0.7% vs. 0.1%, *p* = 0.386), peripheral artery disease history (0.7% vs. 0.6%, *p* = 1.000), and dyslipidaemia (95.0% vs. 95.8%, *p* = 0.655) were found to be identical between patients with and without reinfarction.

### 3.2. Acute Management

The proportion of eligible patients with in-hospital reinfarction who received DAPT and statins at arrival and during hospitalization was lower than that who did not experience reinfarction (90.2% vs. 95.0% (*p* < 0.006) and 89.5% vs. 93.9% (*p* = 0.022), respectively). Regarding revascularization, among patients with reinfarction, 80.0% received acute reperfusion therapy, 73.7% underwent primary PCI, and 8.7% received fibrinolytic therapy; the proportion of those without reinfarction was 85.2%, 83.7%, and 3.6%, respectively. The proportions of ACEI/ARB, *β*-blocker, and anticoagulant use were comparable between patients with and without reinfarction (42.5% vs. 46.7% (*p* = 0.293), 53.6% vs. 53.2% (*p* = 0.293), and 78.4% vs. 79.3% (*p* = 0.799), respectively) (Table 2).

### 3.3. Heart Rates and Myocardial Infarction History Could Be Potential Influential Factors of In-Hospital Reinfarction

Univariate Cox regression showed that age, sex, heart rate, complications within 24 hours, smoking, MI history, HF history, COPD history, RF history, insufficient renal function, stroke, hypertension, diabetes, PCI treatment, DAPT, statins, anaemia, and reduced LVEF differed between patients with and without reinfarction. On the basis of clinical significance and a limited reinfarction population, we entered age, sex, heart rate, smoking, MI history, HF history, COPD history, stroke, hypertension, diabetes, PCI, DAPT, and statin use into Cox regression models. Competing risk analysis showed that patients with a higher heart rate (OR 1.018; 95% CI 1.003 to 1.033) were more susceptible to in-hospital reinfarction, and myocardial infarction history (OR 2.840; 95% CI 1.160 to 6.955) was a risk factor of in-hospital reinfarction independent of hypertension, diabetes, and dyslipidaemia (Table 3).

## 4. Discussion

In this study, 39915 STEMI patients from 150 hospitals throughout China who were registered in the CCC-ACS project were included. Patients with in-hospital reinfarction were characterized in a comprehensive manner. We presented the demographics, clinical condition on admission, medical history, and care during hospitalization of STEMI patients with and without in-hospital reinfarction. In general, patients with reinfarction had advanced age, worse cardiac function, and a history of CHD. A competing risk model showed that a higher heart rate (OR 1.018 (1.003, 1.033)) and myocardial infarction history (OR 2.840 (1.160, 6.955)) could be potential influential factors of in-hospital reinfarction. The study benefited from the large population size and appreciable geographic distribution within China. We think that the data we showed in this study comprehensively fill a gap in the knowledge of the in-hospital reinfarction population in China, though this was a case-control study with low power to predict influential factors.

An elevated heart rate is associated with adverse outcomes in the general population, as well as in patients with hypertension, stable CAD, and chronic HF [27, 28]. In previous studies, an increased resting heart rate was recognized as a risk factor for chronic heart failure [28]. Additionally, among AMI patients without heart failure, patients with lower heart rate or *β*-blocker therapy had a better prognosis [29]. According to this study, the OR of heart rate in the competing risk model was 1.018, meaning that every 10 bpm increase in HR raises the risk of in-hospital reinfarction by 18%, which indicated that heart rate reduction therapy might be beneficial even for patients without heart failure. Physiologically, elevated heart rate increases oxygen demand whilst reducing coronary perfusion time, which impairs coronary blood flow. Deficiency of oxygen supply increases vascular oxidative stress and deteriorates endothelial function which is considered a key event in the development of atherosclerosis and implies a change from the normally predominant release of nitric oxide to that of endothelium-derived contracting factors [30–33]. Hemodynamic factors which are at least defined by the duration/length of the cardiac cycle and are characterized by pulsatility and frequency of mechanical stress may precipitate plaque disruption [34, 35]. To sum up, elevated heart rate potentially promotes both the development of atherosclerosis and the plaque instability. Moreover, a proangiogenic effect of heart rate reduction was displayed using bradycardic pacing in a rabbit model and enhanced vascular endothelial growth factor expression was shown to be critical in bradycardia-induced angiogenesis [36, 37]. We think that beta-blockers and the I(f) channel inhibitor ivabradine could be considered for secondary prevention in STEMI patients with high heart rates. Additional studies need to be performed to draw further conclusions.

Competing risk model analysis indicated that myocardial infarction history was a risk factor of in-hospital reinfarction independent of hypertension, diabetes, and dyslipidaemia. Assumptions could be made that patients with certain lifestyles or genetics were more vulnerable to myocardial infarction. The HMGA1 rs146052672 variant was shown to be associated with myocardial infarction susceptibility [38]. ATG7 DNA sequence variants (DSVs) and single-nucleotide polymorphisms (SNPs) identified in AMI patients may alter the transcriptional activity of the ATG7 gene promoter and change ATG7 levels, contributing to AMI development as a rare risk factor [39]. Similarly, DSVs and SNPs of SIRT5 were suggested to contribute to AMI development as a risk factor [40]. Likewise, people who have a history of circadian rhythm disorders, eating disorders, depression, and so forth were suggested to have a high risk of myocardial infarction [41–43]. More studies need to be carried out to address genetic and environmental determinants of myocardial infarction for better prevention.

## 5. Conclusion

From the data we obtained, we concluded that heart rate and myocardial infarction history might be potential influential factors of in-hospital reinfarction.

## Figures and Tables

**Table 1:** Clinical characteristics of STEMI patients on admission.

Variables	Total (*n* = 39915)	Reinfarction (*n* = 153)	Non-reinfarction (*n* = 39762)	*p* value
Female (%) (*n*)	21.7 (8678)	34 (52)	21.7 (8626)	<0.001
Age (y), mean (SD)	61.65 (12.6)	67.60 (11.1)	61.63 (12.6)	<0.001
BMI, median (quartile)	24.34 (3.63)	23.80 (4.01)	24.34 (3.62)	0.208
SBP (mmHg), mean (SD)	127.11 (23.4)	129.58 (26.1)	127.09 (23.4)	0.242
DBP (mmHg), mean (SD)	77.55 (14.7)	77.52 (15.4)	77.55 (14.7)	0.981
HR (bpm), mean (SD)	77.97 (16.5)	82.94 (17.9)	77.96 (16.5)	0.001
Cardiac shock (%) (*n*)	4.1 (1628)	11.8 (18)	4.0 (1610)	<0.001
Heart failure (%) (*n*)	8.9 (3511)	18.0 (27)	8.9 (3484)	<0.001
Cardiac arrest (%) (*n*)	2.7 (1046)	7.9 (12)	2.6 (1034)	0.001
Killip class (%) (*n*)				<0.001
I	70.6 (26853)	60 (90)	70.6 (26763)	—
II-III	24.8 (9428)	28.0 (42)	24.8 (9386)	—
IV	4.6 (1754)	12.0 (18)	4.6 (1736)	—
Transfer (%) (*n*)	51.6 (20598)	43.1 (66)	51.6 (20532)	0.036
Mortality (%) (*n*)	2.3 (905)	30.7 (47)	2.2 (858)	<0.001
Smoking (%) (*n*)	47.3 (18885)	37.9 (58)	47.3 (18827)	0.002
MI history (%) (*n*)	5.2 (2071)	20.3 (31)	5.1 (2040)	<0.001
PCI history (%) (*n*)	4.5 (1788)	15.0 (23)	4.4 (1765)	<0.001
CABG history (%) (*n*)	0.2 (90)	0.7 (1)	0.2 (89)	0.791
CHD family history (%) (*n*)	2.5 (1008)	1.3 (2)	2.5 (1006)	0.481
Atrial fibrillation (%) (*n*)	1.5 (582)	2.0 (3)	1.5 (579)	0.856
Heart failure history (%) (*n*)	0.9 (378)	5.9 (9)	0.9 (369)	0.001
COPD history (%) (*n*)	1.3 (523)	4.6 (7)	1.3 (516)	0.001
Renal failure (%) (*n*)	1.1 (425)	3.9 (6)	1.1 (419)	0.001
Insufficient renal function (%) (*n*)	22.3 (5818)	37.0 (37)	22.3 (5781)	<0.001
Valvular disease history (%) (*n*)	0.1 (41)	0.7 (1)	0.1 (40)	0.386
Peripheral artery disease (%) (*n*)	0.6 (247)	0.7 (1)	0.6 (246)	1
Stroke (%) (*n*)	8.6 (3447)	14.4 (22)	8.6 (3425)	0.011
Hypertension (%) (*n*)	64.2 (25456)	75.2 (115)	64.1 (25341)	0.004
Diabetes (%) (*n*)	47.0 (17436)	56.4 (79)	47.0 (17357)	0.026
Elevated fasting glucose (%) (*n*)	35.2 (12817)	42.4 (56)	35.2 (12761)	0.082
Elevated HbA1c (%) (*n*)	37.2 (13868)	43.1 (59)	37.2 (13809)	0.157
Dyslipidaemia (%) (*n*)	95.8 (35292)	95.0 (134)	95.8 (35158)	0.655
Elevated LDL (%) (*n*)	86.8 (31222)	86.6 (116)	86.8 (31106)	0.946
Lowered HDL (%) (*n*)	46.2 (16668)	36.3 (49)	46.2 (16619)	0.021
Elevated TG (%) (*n*)	18.6 (6765)	12.6 (17)	18.7 (6748)	0.071
Anaemia (%) (*n*)	19.8 (7262)	32.6 (46)	19.8 (7216)	<0.001
Reduced LVEF (%) (*n*)	31.2 (9637)	50.9 (57)	31.3 (9580)	<0.001

Abbreviations: OR = odds ratio; CI = confidence interval; BMI = body mass index; SBP = systolic blood pressure; DBP = diastolic blood pressure; HR = heart rate; MI = myocardial infarction; PCI = percutaneous coronary intervention; CABG = coronary artery bypass grafting; CHD = coronary heart disease; COPD = chronic obstructive pulmonary disease; LDL = low-density lipoprotein; HDL = high-density lipoprotein; TG = triglyceride.

**Table 2:** Acute management within 24 hours of admission.

Variables	Total (*n* = 39915)	Reinfarction (*n* = 153)	Nonreinfarction (*n* = 39762)	*p*
Fibrinolysis (%) (*n*)	3.6 (1350)	8.7 (11)	3.6 (1339)	0.005
PCI (%) (*n*)	83.7 (30026)	73.7 (84)	83.8 (29942)	0.005
Reperfusion (%) (*n*)	85.2 (34860)	80.0 (88)	85.2 (29616)	0.123
DAPT (%) (*n*)	95.0 (37922)	90.2 (138)	95.0 (37784)	0.006
Aspirin (%) (*n*)	96.0 (38295)	92.2 (141)	96.0 (38154)	0.017
P2Y12 inhibitor (%) (*n*)	96.7 (38591)	93.5 (143)	96.7 (38448)	0.024
Clopidogrel (%) (*n*)	76.0 (30337)	75.8 (116)	76.0 (30211)	0.989
Ticagrelor (%) (*n*)	26.2 (10436)	22.9 (35)	26.2 (10401)	0.354
*β*-Blocker (%) (*n*)	53.3 (21246)	53.6 (82)	53.2 (21164)	0.932
ACEI/ARB (%) (*n*)	46.7 (18636)	42.5 (65)	46.7 (18571)	0.293
Statin (%) (*n*)	93.9 (37482)	89.5 (137)	94.0 (37345)	0.022
GP IIb/IIa receptor blockers (%) (*n*)	38.9 (15503)	35.9 (55)	38.9 (15448)	0.461
Anticoagulants (%) (*n*)	79.3 (31626)	78.4 (120)	79.3 (31506)	0.799

**Table 3:** Adjusted odds ratios from standard Cox analysis and competing risk analysis.

Variables	OR (95% CI)	*p*	Adjusted OR (95% CI)	Adjusted *p*
Age	1.02 (0.994, 1.046)	0.138	1.019 (0.998, 1.041)	0.082
Sex	1.565 (0.785, 3.12)	0.203	1.56 (0.749, 3.248)	0.235
Heart rate	1.019 (1.004, 1.034)	0.015	1.018 (1.003, 1.033)	0.016
Smoking	1.098 (0.564, 2.137)	0.783	1.1 (0.565, 2.142)	0.78
MI history	2.84 (1.204, 6.699)	0.017	2.84 (1.16, 6.955)	0.022
HF history	1.581 (0.336, 7.437)	0.562	1.543 (0.274, 8.691)	0.623
COPD history	2.058 (0.473, 8.96)	0.336	2.041 (0.434, 9.613)	0.367
Stroke	0.929 (0.362, 2.383)	0.878	0.927 (0.359, 2.394)	0.876
Hypertension	1.116 (0.576, 2.164)	0.745	1.114 (0.558, 2.224)	0.76
Diabetes	1.079 (0.602, 1.933)	0.799	1.064 (0.584, 1.936)	0.84
PCI	0.766 (0.387, 1.596)	0.506	0.806 (0.391, 1.661)	0.56
DAPT	2.654 (0.349, 20.197)	0.346	2.665 (0.392, 18.122)	0.316
Statins	0.633 (0.192, 2.092)	0.454	0.648 (0.215, 1.952)	0.441

## Data Availability

As a collaborative initiative of the American Heart Association (AHA) and the Chinese Society of Cardiology (CSC), the CCC-ACS project collected in-hospital data of ACS patients from 150 hospitals all over China. Rationale and design of it have been published previously. (1) Briefly, 150 tertiary hospitals from different geographic and economic regions of China registered in this project. In each hospital, the first 20 to 30 inpatients are consecutively recruited to the study. STEMI was defined strictly complied with the guidelines issued by CSC for diagnosis and management. (2–4) The diagnostic criteria for STEMI were based on chest pain or discomfort, ECG, and measurements of myocardial injury biomarkers. From November 2014 to June 2017, 39915 STEMI inpatients were registered. (1) Hao Y, Liu J, Liu J, et al. Rationale and design of the Improving Care for Cardiovascular Disease in China (CCC) project A national effort to prompt quality enhancement for acute coronary syndrome. Am Heart J. 2016; 179: 107-15. (2) Chinese Society of Cardiology. Guideline for diagnosis and treatment of patients with ST-elevation myocardial infarction 2010. Chin J Cardiol. 2010; 38: 675–690. (3) Chinese Society of Cardiology. 2019 Chinese Society of Cardiology (CSC) guidelines for the diagnosis and management of patients with ST-segment elevation myocardial infarction. Chin J Cardiol. 2019; 47 (10): 766-783. (4) Chinese Society of Cardiology. Guidelines for the management of acute coronary syndromes in patients presenting without persistent ST-segment elevation 2012. Chin J Cardiol. 2012; 40: 353–367.

## Abbreviations

STEMI: ST-segment elevation myocardial infarction
CCC: Improving Care for Cardiovascular Disease in China
ACS: Acute coronary syndrome
MI: Myocardial infarction
COPD: Chronic obstructive pulmonary disease
DAPT: Dual antiplatelet therapy
HR: Heart rate
CVD: Cardiovascular disease
MACE: Major adverse cardiovascular event
AMI: Acute myocardial infarction
AHA: American Heart Association
CSC: Chinese Society of Cardiology
AF: Atrial fibrillation
ECG: Electrocardiogram
CS: Cardiac shock
HF: Heart failure
CA: Cardiac arrest
HbA1c: Glycated haemoglobin A1c
SBP: Systolic blood pressure
DBP: Diastolic blood pressure
LDL-C: Low-density lipoprotein cholesterol
HDL-C: High-density lipoprotein cholesterol
TG: Triglyceride
LVEF: Left ventricular ejection fraction
CHD: Coronary heart disease
PCI: Percutaneous coronary intervention
CABG: Coronary artery bypass graft
PAD: Periphery artery disease
RF: Renal failure
ACEIs: Angiotensin-converting enzyme inhibitors
ARBs: Angiotensin-receptor blockers
SD: Standard deviation
IQR: Interquartile range.
